# Supplementary material for: Investigating the prevalence of cognitive impairment and dementia in the Northern Ireland Cohort for the Longitudinal Study of Ageing (NICOLA): the Harmonised Cognitive Assessment Protocol (HCAP) cross-sectional substudy
Source: BMJ Open. 2024 Jan 31;14(1):e075672. doi: 10.1136/bmjopen-2023-075672 (PMC10831431; doi:10.1136/bmjopen-2023-075672)
Supplement: Author's manuscript [file bmjopen-2023-075672.draft_revisions.pdf]

BMJ Open

BMJ Open is committed to open peer review. As part of this commitment we make the peer review history of every article we publish publicly available.

When an article is published we post the peer reviewers' comments and the authors' responses online. We also post the versions of the paper that were used during peer review. These are the versions that the peer review comments apply to.

The versions of the paper that follow are the versions that were submitted during the peer review process. They are not the versions of record or the final published versions. They should not be cited or distributed as the published version of this manuscript.

BMJ Open is an open access journal and the full, final, typeset and author-corrected version of record of the manuscript is available on our site with no access controls, subscription charges or pay-per-view fees (http://bmjopen.bmj.com).

If you have any questions on BMJ Open's open peer review process please email info.bmjopen@bmj.com

BMJ Open

The Northern Ireland Cohort for the Longitudinal Study of Ageing (NICOLA): The Harmonised Cognitive Assessment Protocol (HCAP) Sub-Study.

Journal:	*BMJ Open*
Manuscript ID	bmjopen-2023-075672
Article Type:	Protocol
Date Submitted by the Author:	15-May-2023
Complete List of Authors:	O'Hara, Leeanne; Queen's University Belfast, Centre for Public Health<br>Neville, Charlotte; Queen's University Belfast, Centre for Public Health<br>Marr, Calum; Queen's University Belfast, Centre for Public Health<br>McAlinden, Michael; Queen's University Belfast, Centre for Public Health<br>Kee, Frank; Queen's University Belfast, Centre for Public Health<br>Weir, David; University of Michigan<br>McGuinness, Bernadette; Queen's University Belfast, Centre for Public Health
Keywords:	Dementia < NEUROLOGY, Aging, PUBLIC HEALTH

SCHOLARONE™
Manuscripts

Title: The Northern Ireland Cohort for the Longitudinal Study of Ageing (NICOLA): The Harmonised Cognitive Assessment Protocol (HCAP) Sub-Study.

Authors: Leeanne O'Hara*, Charlotte Neville, Calum Marr, Michael McAlinden, Frank Kee, David Weir**, Bernadette McGuinness.

Affiliation: Centre for Public Health, Queen's University Belfast, Belfast, UK.

**Survey Research Centre, Institute for Social Research, University of Michigan, Michigan, US.

*Corresponding Author: Centre for Public Health, School of Medicine, Dentistry and Biomedical Sciences, Queen's University Belfast, Institute of Clinical Science, Grosvenor Road, Belfast, BT12 6BJ; Email: l.ohara@qub.ac.uk

Word Count: 4575 (Not including 'References')

Key Words: NICOLA, NICOLA-HCAP, Ageing, Cognition, Dementia, Cognitive Impairment.

For peer review only

Abstract

Introduction

The Northern Ireland Cohort for the Longitudinal Study of Ageing (NICOLA) study is the largest study of ageing in Northern Ireland. The Harmonised Assessment Protocol (HCAP) is a sub-study of NICOLA designed to assess cognitive impairment and dementia in individuals aged 65 and over. The NICOLA-HCAP sub-study is funded by the National Institute on Ageing (NIA) as part of a network for enhancing cross-national research within a worldwide group of population-based, longitudinal studies of ageing, all of which are centred around the US based Health and Retirement Study (HRS).

Methods and Analysis

The NICOLA-HCAP study will draw on the main NICOLA cohort (of 8,283 participants) and randomly sample 1000 participants aged 65 and over to take part in the sub-study. Participants will complete a series of cognitive tests (n=19) via a computer assisted personal interview (CAPI) administered in their home (or alternatively within the research centre) and will be asked to nominate a family member or friend to complete an additional interview of validated instruments to provide information on respondent's prior and current cognitive and physical functioning and whether the individual requires help with daily activities. The objectives of the study are: to investigate the prevalence of dementia and cognitive impairment in NICOLA; harmonise scoring of the NICOLA- HCAP data to the HCAP studies conducted in the Republic of Ireland, the United States of America and England; to explore the validity of dementia estimates; and investigate the risk factors for dementia and cognitive impairment.

Ethics and Dissemination

The study received ethical approval from the Faculty of Medicine, Health and Life Sciences Research Ethics Committee, Queen's University Belfast. The work will provide reliable data for researchers as on study completion, NICOLA-HCAP data will be deposited on the Dementia Platform UK.

For peer review only

Article Summary

Strengths and Limitations of this Study

1. The NICOLA-HCAP sub-study administers the same measures as the HRS (US), the Irish Longitudinal Study of Ageing (TILDA) and the English Longitudinal Study of Ageing (ELSA) HCAP studies, and is part of a wider network of HCAP studies (including, for example, Mexico, India, China, and Europe). HCAP studies will address the need for high-quality data from nationally and internationally representative samples of older men and women and enable cross-country comparisons of dementia prevalence and estimates.

For peer review only - http://bmjopen.bmj.com/site/about/guidelines.xhtml

2. NICOLA-HCAP will enable the identification of possible modifiable risk factors of dementia and can make a significant impact to intervention and prevention.
3. A limitation to the work is that NICOLA-HCAP is currently a cross-sectional study. The aim is to conduct further follow-up studies as per HRS and ELSA.
4. Further, as with any cohort sub-study there is a risk of respondent burden. NICOLA Wave 3 which is due to commence at the end of 2023 will also include measures of cognition. It will therefore be necessary to stage data collection with those who participated in the NICOLA-HCAP sub-study to reduce burden and minimise the risk of respondent familiarity with certain tests.

For peer review only

Introduction

The Northern Ireland Cohort for the Longitudinal Study of Ageing (NICOLA)

The Northern Ireland Cohort for the Longitudinal Study of Ageing (NICOLA) is the largest public health cohort study of ageing in Northern Ireland[1]. In brief, NICOLA was designed to maximise comparability with other well established longitudinal studies of ageing, including the Health and Retirement Study (HRS)[2] in the United States, the Irish Longitudinal Study of Ageing (TILDA)[3] and the English Longitudinal Study of Ageing (ELSA)[4]. The study began in 2013 by recruiting a representative sample of approximately 8500 individuals aged 50 years and over living across Northern Ireland. The first wave included three main components: a Computer-Assisted Personal Interview (CAPI) conducted in the participant's own home; a self-completion questionnaire including a dietary questionnaire; and an objective health assessment. Wave two commenced in 2017 and consisted of a follow-up CAPI and self-completion questionnaire. A refresh of the NICOLA cohort sample also took place at the end of Wave 2 to enable the maintenance and representation of new participants aged 50-54 year olds. A further add-on to Wave 2 was the mailing of a self-completion COVID-19 questionnaire to all study participants in February 2021. It is hoped that Wave 3 will commence later in 2023 and it is the intention that the cohort will continue to be followed up for a period of at least 10 years, pending continued funding. The NICOLA study will enable researchers to gain a better understanding of factors that contribute to health and social outcomes in the older population in Northern Ireland[5].

The NICOLA Harmonised Cognitive Assessment Protocol (HCAP)

The Harmonised Cognitive Assessment Protocol (HCAP) is a sub-study of NICOLA designed to assess dementia and cognitive impairment in a sample of NICOLA participants aged 65 years and older. Funded by the National Institute on Ageing (NIA), the NICOLA-HCAP study is part of a framework for enhancing cross-national research within a group of studies worldwide, all of which are centred on

For peer review only - http://bmjopen.bmj.com/site/about/guidelines.xhtml

the US-based Health and Retirement Study (HRS) [2]. The NICOLA-HCAP study is being carried out at the Centre for Public Health at Queen's University, Belfast. This paper describes the cohort profile HCAP and the findings from the pilot study.

Justification for the HCAP Sub-Study

Dementia and mild cognitive impairment

Dementia is a growing public health concern that currently affects more than 55 million people across the world. There are approximately 10 million new cases diagnosed across the globe annually and it is estimated that by 2050, 152 million people will be living with dementia worldwide[6]. In the UK, dementia is the third most common cause of death for men and the leading cause of death among women[7].

Dementia is a syndrome characterised by the progressive and irreversible deterioration of cognitive functioning that affects an individual's thinking, remembering and reasoning as well as behavioural abilities[8,9]. Dementia is a result of neurodegeneration caused by diseases, such as Alzheimer's disease, vascular dementia, dementia with Lewy bodies, and frontotemporal dementia or a series of strokes. While many cases reflect a mixture of pathologies, Alzheimer's disease (AD) is the most common cause of dementia, accounting for approximately 60-70% of dementia diagnoses[10].

Cognitive impairment is characterised by difficulties in remembering previously acquired knowledge, learning new things, concentrating, or making decisions that affect every day life[12]. Cognitive impairment ranges from mild to severe. People with mild impairment may begin to notice changes in cognitive functions, but still be able to carry out everyday activities. However, severe levels of impairment can lead to loss of the ability to understand the meaning or importance of something and the ability to talk or write, resulting in the inability to live independently. Mild cognitive impairment (MCI) is a condition in which someone has minor problems with their cognitive abilities such as memory or thinking[13]. In MCI, these difficulties are worse than would normally be expected for a healthy person of their age[15]. A person with MCI is more likely to go on to develop dementia at a rate of 10-15% each year[14] and as the 'Baby Boomer' generation pass the age of 65, the number of people living with MCI is expected to jump dramatically[16].

Risk factors for dementia and mild cognitive impairment

There is growing evidence that a large proportion of dementia cases might be delayed or prevented with effective control of modifiable risk factors. While ageing is the strongest known risk factor for

For peer review only - http://bmjopen.bmj.com/site/about/guidelines.xhtml

dementia, it does not exclusively affect older people, with young onset dementia representing nine percent of cases[17]. Other risk factors for dementia include lifestyle factors such as sedentary behaviour[18], smoking[19], excess alcohol consumption[20] , being overweight[21], poor diet[22], high cholesterol[23], high blood sugar levels[24] and uncontrolled blood pressure[25]. Hearing loss, depression, traumatic brain injury and pollution are also noted as potentially modifiable risk factors[26].

Further, an array of socio-demographic factors is associated with an increased risk of dementia including living in areas of socio-economic deprivation[27],[28] and areas with low educational attainment[29]. Loneliness is linked to an increased risk of dementia[30] and social isolation has been shown to increase the risk of dementia by 50 percent[31]. The impact of both of these factors has been exacerbated by the COVID-19 pandemic[51]. The consensus among international experts at the G8 Dementia Summit in 2013 was that increasing physical activity and fruit and vegetable consumption, controlling weight, diabetes and high blood pressure, smoking cessation and avoiding excessive alcohol intake could prevent a substantial proportion of dementia cases[32].

Prevalence and cost of dementia

The current estimates of dementia prevalence in the UK indicate that around 7.1 per cent, representing 1 in every 14 of the population aged 65 years and older are diagnosed with dementia[33]. Similarly, in North America, 10 per cent of individuals aged 65 and over receive a dementia diagnosis[34]. However, a substantial number of cases are not diagnosed, leading to difficulties in estimating true prevalence. There is a pressing need for high-quality data from nationally representative samples of older men and women, hence the need for the HCAP study [34]. Reliable national data on incidence and prevalence of dementia and cognitive impairment are vital for service planning, the prediction of future needs, estimating the costs of dementia care and understanding the impact of these conditions on individuals and their families. There is also a strong research need for robust estimates of MCI and dementia in order to facilitate studies of the aetiology and consequences of these conditions.

Although the number of people with dementia is projected to increase throughout the world because of a well-documented demographic shift towards rising numbers of older adults[47], estimates of future levels are complicated by the assumptions of the underlying methods used to derive them. There is evidence from both England and the US that the incidence of dementia has declined over the past decades[35] but it is rising exponentially in low and middle income countries[48].

For peer review only - http://bmjopen.bmj.com/site/about/guidelines.xhtml

The marked increase in numbers of individuals expected to have dementia has important economic implications. With an estimate of 13 trillion US dollars annually attributed to the condition, an increase in prevalence will have significant economic impact globally[36]. The corresponding economic cost to the UK of caring for people with dementia is estimated to grow from £34.7 billion (2019) to £94.1 billion by 2040[37]. As the population ages, a greater proportion of people in the UK and elsewhere will be living with dementia, and more of the country's resources will be required to care for them.

Policy context and relevance

The importance of dementia has been highlighted with the publication of a national dementia strategy for England[38] and Northern Ireland[39], and in the ex-Prime Minister Cameron's 'Challenge on Dementia' in 2012[40]. The latter emphasised the central role of three issues: raising awareness of dementia and reducing social isolation that people with dementia feel; improving the quality of care; and increasing research. Good quality research generates awareness, shapes policy and encourages service development. However, there is an ongoing need for robust estimates of dementia. Evidence based on data from the NHS Quality and Outcome Framework suggests that 56 per cent of dementia cases in England are not formally diagnosed, with wide variations between areas of the country[41]. Therefore, it is hard to reach a clear consensus about the true dementia prevalence in the UK and elsewhere.

For peer review only

Methods and Analysis

Study Aims and Objectives

The aim of the NICOLA-HCAP study is to collect detailed cognitive data in a subset (n=1000) of the NICOLA cohort using an enhanced harmonised protocol assessment, with measures that are currently administered in the US-based HRS[2]. This will enable estimation of the prevalence of cognitive impairment and dementia among older adults and comparisons of the rates of dementia prevalence in the US and Ireland. The TILDA study are conducting their HCAP study concurrently with NICOLA-HCAP, thus enabling the comparison of rates across Ireland. The findings will increase understanding of the personal, social and economic burden of dementia. The study has five main objectives.

Objective 1: Collect in-depth interview assessments.

The collection of in-depth interview assessments involves the administration of standardised NICOLA-HCAP interviews, both with respondents and their family member or friend ('informant'). The protocol was developed by the HRS HCAP for the international network[52]. The respondent's cognitive ability is measured using a computer-assisted personal interview (CAPI). A family member or friend is asked to complete an additional interview of validated measures to provide information on respondent's prior

For peer review only - http://bmjopen.bmj.com/site/about/guidelines.xhtml

and current cognitive and physical functioning and whether the individual requires help with daily activities, two core clinical criteria required for dementia diagnosis.[52]

Objective 2: Harmonise scoring and investigate the prevalence of dementia and cognitive impairment.

Harmonisation of the scoring of the NICOLA-HCAP data to that of HCAP studies conducted in Ireland (TILDA), the United States (HRS) and England (ELSA) will take place. As the work aims to establish research diagnoses of probable presence or absence of dementia and cognitive impairment, algorithms will be developed to ascertain the probable presence or absence of dementia and cognitive impairment in this sub-sample of NICOLA, in collaboration with colleagues from TILDA, HRS and ELSA. Establishing clear assessments of dementia or MCI prevalence rates in the study will allow the extrapolation of dementia rates from this sub-sample to the rest of the NICOLA population in general. As a result, this information will provide the first estimates of dementia and cognitive impairment from a nationally representative sample in Northern Ireland.

Objective 3: Explore the validity of dementia estimates.

The work will involve the construction of estimates of dementia prevalence for the national populations of Northern Ireland and Ireland harmonised to estimates for the United States and England, having first established the representativeness of the national cohorts, and the development of population weights to account for non-random participation and institutionalisation (care homes). Relationships between the harmonised HCAP data and the more limited cognitive and functioning measures in the core Irish studies will be analysed to establish comparable research diagnoses in the full samples to further expand the data available for epidemiologic study, and identify measures that might be added to core studies to improve cognitive assessment going forward.

Objective 4: Investigate the risk factors for dementia and cognitive impairment

Preliminary analyses will be conducted to: a) Investigate potential causal pathways from cardiovascular instability, cerebral microvascular disease and sensory impairment to dementia using objective measures from the TILDA and NICOLA health assessments, including neuroimaging, near-infrared spectroscopy, hearing, vision, retinal photography, and multi-sensory integration technologies and using polygenic scores to capture genetic differences; b) Use cross-country analyses to investigate how stress and long-term exposure to conflict in a unique cohort of older adults affects cognitive aging and physiological ageing and methylation / epigenetic patterns in known risk loci related to traumatic stress; c) Establish potentially modifiable risk factors for primary and secondary prevention of dementia.

For peer review only - http://bmjopen.bmj.com/site/about/guidelines.xhtml

Objective 5: Provide reliable data to researchers.

Data from NICOLA-HCAP will be provided to the research community to complement existing public data resources from these studies and support epidemiological research being conducted elsewhere.

Study Design

The NICOLA-HCAP assessment is comprised of two main parts: a face-to-face interview with the participant, and a shorter interview, with a family member or friend, nominated by the participant (the 'informant').

Participant interview

The participant interview is administered face-to-face using a computer assisted personal interview (CAPI) formatted using Epi Info software package[42]. The interview takes place in the participant's home, however, the option of attending the Centre for Public Health at Queen's University Belfast or the Clinical Translational Research and Innovation Centre (C-TRIC) based in Derry/Londonderry is offered to those who prefer not to have a researcher visit them at home. The interview is approximately one hour in duration and consists of nineteen different measures of memory, language, attention and other aspects of cognitive ability. The Centre for Epidemiologic Studies Depression Scale (CES-D) is administered as an assessment of mental wellbeing to adjust for the impact of depressive symptoms on cognitive test performance (see Table 1). The majority of measures are completed verbally by the participant, with the researcher recording answers on the laptop, while some other measures are completed by the participant using paper and pencil (due to nature of the test and licencing restrictions). Interviews with participants are audio recorded (only with participants' consent) on the laptop for internal quality control processes. All participants are offered a gift voucher (20 GBP) in appreciation of their participation in the study.

For peer review only

For peer review only - http://bmjopen.bmj.com/site/about/guidelines.xhtml

Table 1: Participant CAPI measures

**MEASURES IN THE PARTICIPANT INTERVIEW**
Mini-Mental State Examination (MMSE) [49]
HRS Telephone Interview for Cognitive Status (HRS-TICS)
CERAD Word List Learning and Recall – Immediate
Retrieval Fluency
Letter Cancellation
Backward Count
10/66 Respondent
CERAD Delayed Recall
Wechsler Memory Scale IV – Immediate
CERAD Word List Recognition
Constructional Praxis – Immediate
Symbol-Digit Modalities Test
Constructional Praxis – Delayed
Wechsler Memory Scale IV – Delayed
Wechsler Memory Scale IV – Recognition
Number Series
Raven's Standard Progressive Matrices
Trail Making
CES-D Depressive Symptoms

CES-D, Centre for Epidemiological Studies Depression Scale
CERAD, Consortium to establish a registry for Alzheimer's disease

Family or friend (informant) interview

During the home interview, the participant is asked to nominate a family member or friend (the 'informant') who has known them for some years and whom they think would be willing to answer some questions about the participant and their daily activities. The family or friend interview includes questions relating to changes in the participant's cognitive abilities over time, and activities the participant carries out inside or outside the home (see Table 2). As with the participant interview, the family or friend interview is also administered via CAPI in Epi Info[42] and is audio recorded for quality control purposes. The interview lasts approximately twenty minutes and is administered at the participant's home if the family member or friend is present at the time of the participant interview. If they are not present, the family member or friend is contacted by telephone and the interview is completed over the phone at a time convenient to them. Alternatively, the family member or friend can request a paper version of the questionnaire to be posted to their home or they can complete the questionnaire online (based on Qualtrics user platform[43]) whereby a link to the survey is emailed or texted to their mobile phone.

For peer review only - http://bmjopen.bmj.com/site/about/guidelines.xhtml

Table 2: Informant CAPI measures

MEASURES IN THE INFORMANT INTERVIEW
1. Family or friend demographics
2. Jorm IQCODE
3. Blessed dementia rating scale - Part 2
4. HRS Activities questionnaire
5. CSI- D – Cognitive Activities
6. 10/66 Informant
7. Blessed dementia rating scale - Part 1

IQCODE, informant questionnaire on cognitive decline in the elderly
CSI-D, Community Screening Instrument for Dementia

Sample

The NICOLA-HCAP sample is drawn from core members of the NICOLA cohort. The aim is to recruit 1000 participants who took part in the NICOLA Wave 2 CAPI (end date December 2019), aged 65 years or over at the start of HCAP fieldwork. Both participant and informant interviews are conducted with participants who are residing in private residential accommodation in Northern Ireland. Those who were in institutionalised care i.e. residential homes or nursing homes were excluded from participating in NICOLA and therefore will not be included in NICOLA-HCAP. Individuals are randomly selected and the sample is stratified to include participants who live alone (single household) and participants who do not live alone (multiple household). Participants in multiple households who are initially ineligible but become eligible later (typically spouses who turn 65 after the onset of the study) are also able to take part. An 80 per cent response rate is expected to the NICOLA-HCAP, which implies a selection rate of about 1 in 3 to achieve the target of 1,000 participants. Individuals will be excluded from the study if they are unable to complete the written tasks included in the NICOLA-HCAP assessment (due to reasons such as sensory impairment, e.g. hearing loss, visual impairment) or if they are not fluent in English.

Informants are nominated by participants and can be a family member, friend or carer. The researcher discusses the nomination with the participant once they have agreed to take part in the interview, and recommends that the nominated person is someone who knows the participant well and sees them frequently enough to answer questions about their daily life, as well as being someone who has known them for a number of years. The recommendation is that the nominated person is a family member or friend rather than someone who is employed to be with the participant, such as a carer, however, if a family member or friend is not an option and the employed person has known the participant for long enough, they can fulfil this role.

For peer review only

Proposed statistical methods

Sample weights will be applied as per the HRS protocol adjusting for differential sampling weights by race/ethnicity, age, cognitive ability and for differential participation by demographic factors such as education, socio-economic status etc.

The use of the one-hour battery of identical tests, twenty minutes of informant interview, and similar methods of administration, provides a strong foundation for harmonisation. Subtle differences in scoring can produce disparities, so there will be extensive comparison across studies using audio recordings, participant drawings, and other records to ensure comparability. Led by HRS, statistical procedures such as tests for factor invariance will be used to determine if there are any specific inconsistencies in tests between countries. Similar analyses will be carried out with the informant reports. Based on this determination HRS will construct the final best algorithm for diagnoses that will subsequently be used in the proposed pooled analyses of the four studies (HRS, ELSA, TILDA, NICOLA).

Staff Training

Fieldwork staff working on the NICOLA-HCAP study includes both Queen's University research staff and dementia trained clinical research nurses from the Northern Ireland Clinical Research Network (NICRN). All staff received face-to-face training delivered in two phases. Phase one focused on study orientation and the development of the Standard Operating Procedures (SOP). In brief, sessions with fieldwork staff included an introduction to the research aims and objectives; study design; staffing roles; recruitment procedures and pre-visit protocols; consent procedures and capacity to consent protocols; set up of Epi Info software[42] (for data capture – participant CAPI and informant CAPI) and the upload of data to secure servers. Phase two focused on the administration of cognitive tests and informant measures. The Survey Research Operations (SRO) team based in the Survey Research Centre in the University of Michigan developed training videos and scripts for in-house training. Fieldwork staff met in the first instance as a full team to review the videos and practice administration using the CAPI software. This was followed by practice administration using a buddy system. This involved fieldwork staff working in pairs, with one member of staff adopting the role of the participant and the other the interviewer (and vice versa). As training for TILDA-HCAP was running consecutively with NICOLA-HCAP, joint TILDA-NICOLA meetings were held with the SRO and HRS team to address any queries relating to test administration and scoring. The HRS team subsequently developed a scoring protocol to ensure consistency across studies.

NICOLA-HCAP Dress Rehearsal

For peer review only - http://bmjopen.bmj.com/site/about/guidelines.xhtml

The pilot dress rehearsal mirrored the ELSA-HCAP dress rehearsal[44] and was used to inform the mainstage survey. The main aim of the pilot was to implement the study as per the NICOLA-HCAP study protocol and to identify any areas that needed to be revised or improved in relation to the study design, questionnaire format and content, data collection, data upload processes and materials prior to the main phase and the SOP could be amended accordingly. The pilot dress-rehearsal was conducted between the 6th January 2022 and 25th January 2022.

Pilot Design

The dress rehearsal aimed to recruit 15 participants and 15 family or friend members to take part in the respective interviews. Following each interview, the research nurses respectively completed an interview feedback form that included sections for feedback on both fieldwork procedures and in-house processes. In brief, the fieldwork feedback included information on personal protective equipment (PPE) requirements and any perceived impact on study administration; participant information and consent processes; CAPI and paper administrations; and participant experience. Feedback on the in-house processes centred on the administrative procedures; pre-visit protocols; use of secure servers and the efficiency of the process required to carry out data uploads of participant and family or friend interviews; data storage and any additionally required processes to support the smooth delivery of the project.

Pilot Sample

A total of 14 participant interviews and 14 informant interviews were achieved within the timeframe for the pilot study. The pilot dress rehearsal sample was selected from the NICOLA Healthy Ageing Research Advisory panel which included both NICOLA participants (n=5) and volunteers from AgeNI[45] (n=9) along with their respective family member or friend. Members of the Advisory Panel understood that their role was to test the design, content and materials of the proposed NICOLA-HCAP interview. Ethical approval for the pilot was obtained from the Faculty of Medicine, Health and Life Sciences Research Ethics Committee, Queen's University Belfast.

Pilot Revisions

Feedback obtained from fieldwork staff informed a number of study revisions or improvements that were integrated into the SOP and implemented for the main study. The pilot identified the following key areas for improvement.

For peer review only - http://bmjopen.bmj.com/site/about/guidelines.xhtml

Area one: Pre-Visit Documentation. Fieldwork staff reflected on the complexities of test administration and the transition between the CAPI and paper versions of tests. Pre-visit protocols were revised to include preparation processes (i.e. folders with test papers organised in order of test administration and participant identification numbers pre-added to documentation). Staff also preferred a manual timer for tests rather than the built-in CAPI version.

Area two: Test Administration. Queries were raised in relation to the logistics of some test administration. Fieldwork staff also questioned how tests were scored and entered into the Epi Info software. All queries were resolved with the HRS and TILDA teams.

Area three: Study Administration Processes. Additional administration databases were set up to ease the flow of recruitment and home visit bookings. In addition, the SOP was amended to update in-house filing and the secure storage of data.

Ethics and Dissemination

The main study received ethical approval from the Faculty of Medicine, Health and Life Sciences Research Ethics Committee, Queen's University Belfast[50]. Participants and informants provided written informed consent prior to participation in the study. The NICOLA-HCAP main study commenced on 7th February 2022 with fieldwork due to complete in June 2023. The data from NICOLA-HCAP is currently held in the data repository within the Centre for Public Health at Queen's University Belfast and post study completion, all NICOLA-HCAP data will also be deposited on the Dementias Platform UK (DPUK) [11]. NICOLA Wave 1 and Wave 2 data are also maintained and stored at a data repository within the Centre for Public Health, Queen's University Belfast. Information on how to access NICOLA data and details regarding the data that is currently available in NICOLA can be found on the study website[46].

Acknowledgements

The authors would like to acknowledge the participation of members from the NICOLA Healthy Ageing Research Advisory panel in the NICOLA-HCAP pilot study. Your participation and feedback on the process was invaluable to the study. The authors are also grateful to the participants from the NICOLA study, and the wider NICOLA team for their continued support and assistance and would like to thank the research nurses, clerical staff and computer technicians for their time, dedication, professionalism and contribution to the NICOLA-HCAP study. The authors alone are responsible for the interpretation of the data and any views or opinions presented are solely those of the authors and do not necessarily represent those of the NICOLA study team.

For peer review only - http://bmjopen.bmj.com/site/about/guidelines.xhtml

Author contributions

FK, IY (NICOLA) and BM (NICOLA-HCAP) were responsible for study concept and design. LO, CN, CM, MM, BM prepared the initial drafts of the manuscript, LO carried out subsequent revisions. All authors contributed to the critical revision of the manuscript, proof reading and the approval of the final version.

Funding

The Northern Ireland Cohort for the Longitudinal Study of Ageing Harmonised Cognitive Assessment Protocol (NICOLA-HCAP) is funded by the National Institute on Aging (NIA; Grant number (GN) SUBK00010180). The NICOLA study (Waves 1 and 2) is funded by the Atlantic Philanthropies (GN – 18107), the Economic and Social Research Council (GN -ES/L0084559/1); the UKCRC Centre for Excellence for Public Health Northern Ireland; Northern Ireland Health and Social Care Research and Development Division of the Public Health Agency (GN - STL/4717/12); the Wellcome Trust/Wolfson Foundation, the Centre for Ageing Research and Development Ireland, Queen's University Belfast and the Office of the First Minister and Deputy First Minister Northern Ireland.

Competing Interests.

None declared.

For peer review only

For peer review only - http://bmjopen.bmj.com/site/about/guidelines.xhtml

References

[1]Neville C*, Burns F*, Cruise S, Scott A, O'Reilly D, Kee F, Young I. Cohort Profile: the Northern Ireland Cohort for the Longitudinal Study of Ageing (NICOLA). International Journal of Epidemiology. 2023. Doi: doi: 10.1093/ije/dyad026. Epub ahead of print. PMID: 37011634.

[2]Health and Retirement Study (HRS). https://hrsonline.isr.umich.edu/ (14th March 2023, date last accessed).

[3] Irish Longitudinal Study on Ageing (TILDA). https://tilda.tcd.ie/ (14th March 2023, date last accessed).

[4] English Longitudinal Study of Ageing (ELSA). https://www.elsa-project.ac.uk/ (14th March 2023, date last accessed).

[5]Cruise S, Kee F (Eds). Early key findings from a study of older people in Northern Ireland: The NICOLA Study. Belfast, UK: Queen's University Belfast, 2017. https://www.qub.ac.uk/sites/NICOLA/ReportsandNewsletters/. (14th March 2023, date last accessed)

[6]World Health Organisation. Dementia. https://www.who.int/news-room/fact-sheets/detail/dementia (14th March 2023, date last accessed).

[7]Office for National Statistics. https://www.ons.gov.uk/peoplepopulationandcommunity/healthandsocialcare/causesofdeath/articles/leadingcausesofdeathuk/2001to2018 (14th March 2023, date last accessed).

[8]National Institute on Aging. What is Dementia? Symptoms, Types and Diagnosis. https://www.nia.nih.gov/health/what-is-dementia (14th March 2023, date last accessed).

[9] White S, Griffiths R, Baxter M, Beanland T, Cross J, Dhesi J, Docherty A.B, Foo I, Jolly G, Jones J, Moppett I.K, Plunkett E, Sachdev K. Guidelines for the peri-operative care of people with dementia. Anaesthesia. 2019; 74: 271-273.

[10]World Health Organisation. Dementia.https://www.who.int/news-room/fact-sheets/detail/dementia (14th March 2023, date last accessed).

[11] Dementias Platform UK. https://www.dementiasplatform.uk/. (14th March 2023, date last accessed).

[12] Bassett S.S, & Folstein M.F. Cognitive impairment and functional disability in the absence of psychiatric diagnosis. Psychol Med. 1991; 21(1): 77-84.

[13] Alzheimer's Society. https://www.alzheimers.org.uk/about-dementia/types-dementia/mild-cognitive-impairment-mci (14th March 2023, date last accessed).

[14] Mayo Clinic. https://www.mayoclinic.org/diseases-conditions/mild-cognitive-impairment/symptoms-causes/syc-20354578 (14th March 2023, date last accessed).

[15] Petersen C.P, et al. Cognitive Impairment: Clinical Characterization and Outcome. Arch Neurol. 1999; 56: 303-308.

For peer review only - http://bmjopen.bmj.com/site/about/guidelines.xhtml

[16] Alzheimer's Disease Facts and Figures https://www.cdc.gov/aging/pdf/cognitive_impairment/cogimp_poilicy_final.pdf (01 March 2023, date last accessed).

[17] World Health Organisation. Dementia. https://www.who.int/news-room/fact-sheets/detail/dementia (14th March 2023, date last accessed).

[18] Yan S, Wennning F, Wang C, Mao J, Bing L, Zou L, Lv C. Association between sedentary behaviour and the risk of dementia: A systematic review and meta-analysis. Transitional Psychiatry. 2020; 10; 112. https://doi.org/10.1038/s41398-020-0799-5.

[19] Choi D, Choi S, Park S M. Effect of smoking cessation on the risk of dementia: a longitudinal study. Annals of Clinical and Translational Neurology. 2018; 5 (10); 1192-1199. doi: 10.1002/acn3.633.

[20] Wiegmann C, Mick I, Brandl E J, Heinz A, Gutwinski S. Alcohol and Dementia - What is the link? A systematic review. Neuropsychiatric Disease and Treatment. 2020; 16; 87-99.

[21] Ma Y, Ajnakina O, Steptoe A, Cadar D. Higher risk of Dementia in English older individuals who are overweight or obese. International Journal of Epidemiology. 2020; 1353-1365. doi: 10.1093/ije/dyaa099.

[22] de Crom T O E, Mooldijk S S, Ikram M K, Ikram M A, Voortman T. MIND diet and the risk of dementia: a population-based study. Alzheimer's Research and Therapy. 2022 14; 8. https://doi.org/10.1186/s13195-022-00957-1.

[23] Varma V R, Wang Y, An Y, Varma S, Bigel M, Doshi J, et al. Bile acid synthesis, modulation and dementia: A metabolomic, transcriptomic, and pharmacoepidemiologic study. PLOS Medicine. 2021; 18 (5). doi:10.1371/journal.pmed.1003615.

[24] Crane PK, Walker R, Hubbard, R A, Li G, Nathan D M, et al. Glucose levels and risk of dementia. N Engl J Med. 2013; 369; 540. DOI: 10.1056/NEJMoa1215740

[25] Lee C J, Lee Ji-L, Han K, Kim D H, Cho H, Kim K J, et al. Blood pressure levels and risks of dementia: a Nationwide study of 4.5 million people. Hypertension. 2022; 79 (1); 218-229.

[26] Livingstone G, Huntley J, Sommerlad A, Ames D, Ballard C, Banerjee S, et al. Dementia prevention, intervention and care: 2020 report of the Lancet Commission. The Lancet Commissions. 2020; 396 (10248); 413-446.

[27] Jitlal M, Amirthalingam G N K, Karania T, Parry E, Neligan A, et al. The Influence of Socioeconomic Deprivation on Dementia Mortality, Age at Death, and Quality of Diagnosis: A Nationwide Death Records Study in England and Wales 2001–2017. J Alzheimers Dis. 2021; 81 (1) 321 – 328. doi: 10.3233/JAD-210089.

[28] McMichael A J, Zafeiridi E, Passmore P, Cunningham E L, McGuinness B. Factors associated with mortality including nursing home transitions: A retrospective analysis of 25,418 people prescribed

For peer review only

anti-dementia drugs in Northern Ireland. J Alzheimer's Disease. 2020; 73; 1233-1242. DOI 10.3233/JAD-190751.

[29] Takasugi T, Tsuji T, Hanazato M, Miyaguni, Y, et al. Community-level educational attainment and dementia: a 6-year longitudinal multilevel study in Japan. BMC Geriatrics. 2021; 21:661. https://doi.org/10.1186/s12877-021-02615-x.

[30] Lara E, Martín-María N, De la Torre-Luque A, Koyanagi A, Vancampfort D, Izquierdo A, et al. Does loneliness contribute to mild cognitive impairment and dementia? a systematic review and meta-analysis of longitudinal studies. *Ageing Res. Rev.* 52, 7–16. doi: 10.1016/j.arr.2019.03.002

[31] National Academies of Sciences, Engineering, and Medicine. *Social Isolation and Loneliness in Older Adults: Opportunities for the Health Care System*. 2020. Washington, DC: The National Academies Press

[32]Department of Health and Social Care. https://www.gov.uk/government/publications/g8-dementia-summit-global-action-against-dementia/g8-dementia-summit-global-action-against-dementia-11-december-2013 (14th March 2023, date last accessed)

[33]Alzheimer's Society https://www.alzheimers.org.uk/about-us/policy-and-influencing/dementia-uk-report (14th March 2023, date last accessed)

[34] Manly, J.J., Jones, R.N., Langa, K.M., Ryan, L.H., Levine, D.A., McCammon, R., Heeringa, S.G. and Weir, D., 2022. Estimating the prevalence of dementia and mild cognitive impairment in the US: the 2016 Health and Retirement Study harmonized cognitive assessment protocol project. *JAMA neurology*, *79*(12), pp.1242-1249.

[35] Matthews F.E. et al. A two decade dementia incidence comparison from the Cognitive Function and Ageing Studies 1 and 11. Nature Communications. 2016; 7: 11398. https://doi.org/10.1038/ncomms11398

[36] Livingstone G, Huntley J, Sommerlad A, Ames D, Ballard C, Banerjee S, et al. Dementia prevention, intervention and care: 2020 report of the Lancet Commission. The Lancet Commissions. 2020; 396 (10248); 413-446.

[37]Alzheimer's Society. https://www.alzheimers.org.uk/about-us/policy-and-influencing/dementia-scale-impact-numbers (14th March 2023, date last accessed).

[38]Department of Health and Social Care. https://www.gov.uk/government/publications/living-well-with-dementia-a-national-dementia-strategy. (14th March 2023, date last accessed).

[39]Department of Health. https://www.health-ni.gov.uk/publications/improving-dementia-services-northern-ireland-regional-strategy (14th March 2023, date last accessed).

[40]Department of Health and Social Care. https://www.gov.uk/government/publications/prime-ministers-challenge-on-dementia (14th March 2023, date last accessed).

For peer review only - http://bmjopen.bmj.com/site/about/guidelines.xhtml

For peer review only

[41]NHS.https://digital.nhs.uk/data-and-information/publications/statistical/nhs-outcomes-framework (14th March 2023, date last accessed).

[42] Centres for Disease Control and Prevention. https://www.cdc.gov/epiinfo/index.html (14th March 2023, date last accessed).

[43] Qualtrics XM. https://www.qualtrics.com/uk/ (14th March 2023, date last accessed).

[44] Cadar D, Abell J, Matthews FE, Brayne C, David Batty G, Llewellyn DJ, et al. Cohort Profile Update: The Harmonised Cognitive Assessment Protocol Sub-study of the English Longitudinal Study of Ageing (ELSA-HCAP). Int J Epidemiol. 2021;50(3):725-6i. DOI:10.1093/ije/dyaa227 pmcid:PMC8271185

[45]AgeNI. https://www.ageni.org/ (14th March 2023, date last accessed).

[46] NICOLA. https://www.qub.ac.uk/sites/NICOLA/Informationforresearchers/ (14th March 2023, date last accessed).

[47] World Health Organisation. https://www.who.int/news-room/fact-sheets/detail/ageing-and-health (14th March 2023, date last accessed).

[48] Pedroza P. et al. Global and regional spending on dementia care from 2000-2019 and expected future health spending scenarios from 2020-2050: An economic modelling exercise. The Lancet. 2022; 45: 101337. DOI: https://doi.org/10.1016/j.eclinm.2022.101337.

[49] MMSE Mini-Mental State Examination. https://www.parinc.com/Products/Pkey/237. (14th March 2023, date last accessed).

[50] Faculty of Medicine, Health and Life Sciences Research Ethics Committee, Queen's University Belfast https://www.qub.ac.uk/Research/Governance-ethics-and-integrity/Ethics/FacultyResearchEthicsCommittees/ (14th March 2023, date last accessed).

[51] Belfast City Council. Age Friendly Belfast Plan 2022-2026. https://yoursay.belfastcity.gov.uk/age-friendly-plan-2022-26. 2022. (14th March 2023, date last accessed).

[52] Langa KM, Ryan LH, McCammon R, Jones RN, Manly JJ, Levine DA, Sonnega A, Farron M & Weir D. The Health and Retirement Study Harmonized Cognitive Assessment Protocol (HCAP) Project: Study Design and Methods. Neuroepidemiology. 2021; 54(1):64-74. DOI: 10.1159/000503004.

For peer review only

Dr Leeanne O’Hara

Centre for Public Health,

Queen’s University Belfast,

Belfast,

Northern Ireland.

BT12 6BJ

15th May, 2023

Dear Sir/Madam,

Please find attached submission to the BMJ Open for review. This paper is a protocol for the Northern Ireland Cohort for the Longitudinal Study of Ageing (NICOLA): The Harmonised Cognitive Assessment Protocol (HCAP) Sub-Study. The word count for this paper is 4575 (excluding references). I look forward to hearing from you in due course.

Yours sincerely,

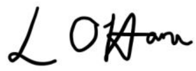

Dr Leeanne O’Hara

For peer review only

BMJ Open

Investigating the prevalence of Cognitive Impairment and Dementia in the Northern Ireland Cohort for the Longitudinal Study of Ageing (NICOLA): The Harmonised Cognitive Assessment Protocol (HCAP) Cross-sectional Sub-study.

Journal:	*BMJ Open*
Manuscript ID	bmjopen-2023-075672.R1
Article Type:	Protocol
Date Submitted by the Author:	21-Dec-2023
Complete List of Authors:	O'Hara, Leeanne; Queen's University Belfast, Centre for Public Health<br>Neville, Charlotte; Queen's University Belfast, Centre for Public Health<br>Marr, Calum; Queen's University Belfast, Centre for Public Health<br>McAlinden, Michael; Queen's University Belfast, Centre for Public Health<br>Kee, Frank; Queen's University Belfast, Centre for Public Health<br>Weir, David; University of Michigan<br>McGuinness, Bernadette; Queen's University Belfast, Centre for Public Health
**Primary Subject Heading**:	Public health
Secondary Subject Heading:	Geriatric medicine, Global health
Keywords:	Dementia < NEUROLOGY, Aging, PUBLIC HEALTH

SCHOLARONE™
Manuscripts

Title: Investigating the prevalence of Cognitive Impairment and Dementia in the Northern Ireland Cohort for the Longitudinal Study of Ageing (NICOLA): The Harmonised Cognitive Assessment Protocol (HCAP) Cross-sectional Sub-study.

Authors: Leeanne O'Hara*, Charlotte Neville, Calum Marr, Michael McAlinden, Frank Kee, David Weir**, Bernadette McGuinness.

Affiliation: Centre for Public Health, Queen's University Belfast, Belfast, UK.

**Survey Research Centre, Institute for Social Research, University of Michigan, Michigan, US.

*Corresponding Author: Centre for Public Health, School of Medicine, Dentistry and Biomedical Sciences, Queen's University Belfast, Institute of Clinical Science, Grosvenor Road, Belfast, BT12 6BJ; Email: l.ohara@qub.ac.uk

Word Count: 4965 (Not including 'References')

Key Words: NICOLA, NICOLA-HCAP, Ageing, Cognition, Dementia, Cognitive Impairment.

For peer review only

Abstract

Introduction

The Northern Ireland Cohort for the Longitudinal Study of Ageing (NICOLA) study is the largest study of ageing in Northern Ireland. The Harmonised Assessment Protocol (HCAP) is a sub-study of NICOLA designed to assess cognitive impairment and dementia in individuals aged 65 and over. The NICOLA-HCAP sub-study is funded by the National Institute on Ageing (NIA) as part of a network for enhancing cross-national research within a worldwide group of population-based, longitudinal studies of ageing, all of which are centred around the US based Health and Retirement Study (HRS).

Methods and Analysis

The NICOLA-HCAP study will draw on the main NICOLA cohort (of 8,283 participants) and randomly sample 1000 participants aged 65 and over to take part in the sub-study. Participants will complete a series of cognitive tests (n=19) via a computer assisted personal interview (CAPI) administered in their home (or alternatively within the research centre) and will be asked to nominate a family member or friend to complete an additional interview of validated instruments to provide information on respondent's prior and current cognitive and physical functioning and whether the individual requires help with daily activities. The objectives of the study are: to investigate the prevalence of dementia and cognitive impairment in NICOLA; harmonise scoring of the NICOLA- HCAP data to the HCAP studies conducted in the Republic of Ireland, the United States of America and England; to explore the validity of dementia estimates; and investigate the risk factors for dementia and cognitive impairment.

Ethics and Dissemination

The study received ethical approval from the Faculty of Medicine, Health and Life Sciences Research Ethics Committee, Queen's University Belfast. We will provide data from the Northern Irish HCAP to the research community via data repositories such as the Dementias Platform UK (DPUK) and Gateway to Global Aging to complement existing public data resources and support epidemiologic research by others. Findings will also be disseminated through peer reviewed publications and at international conferences.

For peer review only

Article Summary

Strengths and Limitations of this Study

1. The strength of NICOLA-HCAP is that the sub-study administers the same measures as the HRS (US), the Irish Longitudinal Study of Ageing (TILDA) and the English Longitudinal Study of Ageing (ELSA) HCAP studies, and is part of a wider network of HCAP studies (including, for

For peer review only - http://bmjopen.bmj.com/site/about/guidelines.xhtml

example, Mexico, India, China, and Europe). Further, the measures are all objective measures of cognitive health.

2. The scope and detail of data collected within the NICOLA study allow for in depth analysis and understanding of factors that affect cognition.
3. A limitation to the work is that NICOLA-HCAP is currently a cross-sectional study. The aim is to conduct further follow-up studies as per HRS and ELSA.
4. As with any cohort sub-study there is also a risk of respondent burden. NICOLA Wave 3 which is due to commence at the end of 2023 will also include measures of cognition. It will therefore be necessary to stage data collection with those who participated in the NICOLA-HCAP sub-study to reduce burden and minimise the risk of respondent familiarity with certain tests.

For peer review only

Introduction

The Northern Ireland Cohort for the Longitudinal Study of Ageing (NICOLA)

The Northern Ireland Cohort for the Longitudinal Study of Ageing (NICOLA) is the largest public health cohort study of ageing in Northern Ireland [1]. In brief, NICOLA was designed to maximise comparability with other well established longitudinal studies of ageing, including the Health and Retirement Study (HRS) [2] in the United States, the Irish Longitudinal Study of Ageing (TILDA) [3] and the English Longitudinal Study of Ageing (ELSA) [4]. The study began in 2013 by recruiting a representative sample of approximately 8500 individuals aged 50 years and over living across Northern Ireland. The first wave included three main components: a Computer-Assisted Personal Interview (CAPI) conducted in the participant's own home; a self-completion questionnaire including a dietary questionnaire; and an objective health assessment. Wave two commenced in 2017 and consisted of a follow-up CAPI and self-completion questionnaire. A refresh of the NICOLA cohort sample also took place at the end of Wave 2 to enable the maintenance and representation of new participants aged 50-54 year olds. A further add-on to Wave 2 was the mailing of a self-completion COVID-19 questionnaire to all study participants in February 2021 [5]. It is hoped that Wave 3 will commence later in 2023 and it is the intention that the cohort will continue to be followed up for a period of at least 10 years, pending continued funding. The NICOLA study will enable researchers to gain a better understanding of factors that contribute to health and social outcomes in the older population in Northern Ireland [6].

The NICOLA Harmonised Cognitive Assessment Protocol (HCAP)

For peer review only - http://bmjopen.bmj.com/site/about/guidelines.xhtml

The Harmonised Cognitive Assessment Protocol (HCAP) is a sub-study of NICOLA designed to assess dementia and cognitive impairment in a sample of NICOLA participants aged 65 years and older. Funded by the National Institute on Ageing (NIA), the NICOLA-HCAP study is part of a framework for enhancing cross-national research within a group of studies worldwide, all of which are centred on the US-based Health and Retirement Study (HRS) [2]. The NICOLA-HCAP study is being carried out at the Centre for Public Health at Queen's University, Belfast. This paper describes the cohort profile HCAP and the findings from the pilot study.

Justification for the HCAP Sub-Study

Dementia and mild cognitive impairment

Dementia is a growing public health concern that currently affects more than 55 million people across the world. There are approximately 10 million new cases diagnosed across the globe annually and it is estimated that by 2050, 152 million people will be living with dementia worldwide [6]. In the UK, dementia is the third most common cause of death for men and the leading cause of death among women [7].

Dementia is a syndrome characterised by the progressive and irreversible deterioration of cognitive functioning that affects an individual's thinking, remembering and reasoning as well as behavioural abilities [8; 9]. Dementia is a result of neurodegeneration caused by diseases, such as Alzheimer's disease, vascular dementia, dementia with Lewy bodies, and frontotemporal dementia or a series of strokes. While many cases reflect a mixture of pathologies, Alzheimer's disease (AD) is the most common cause of dementia, accounting for approximately 60-70% of dementia diagnoses [10].

Cognitive impairment is characterised by difficulties in remembering previously acquired knowledge, learning new things, concentrating, or making decisions that affect everyday life [11]. Cognitive impairment ranges from mild to severe. People with mild impairment may begin to notice changes in cognitive functions, but still be able to carry out everyday activities. However, severe levels of impairment can lead to loss of the ability to understand the meaning or importance of something and the ability to talk or write, resulting in the inability to live independently. Mild cognitive impairment (MCI) is a condition in which someone has minor problems with their cognitive abilities such as memory or thinking [12]. In MCI, these difficulties are worse than would normally be expected for a healthy person of their age [13]. A person with MCI is more likely to go on to develop dementia at a rate of 10-15% each year [14] and as the 'Baby Boomer' generation pass the age of 65, the number of people living with MCI is expected to jump dramatically [15].

For peer review only - http://bmjopen.bmj.com/site/about/guidelines.xhtml

Risk factors for dementia and mild cognitive impairment

There is growing evidence that a large proportion of dementia cases might be delayed or prevented with effective control of modifiable risk factors. While ageing is the strongest known risk factor for dementia, it does not exclusively affect older people, with young onset dementia representing nine percent of cases [16]. Other risk factors for dementia include lifestyle factors such as sedentary behaviour [17], smoking [18], excess alcohol consumption [19] , being overweight [20], poor diet [21], high cholesterol [22], high blood sugar levels [23] and uncontrolled blood pressure [24]. Hearing loss, depression, traumatic brain injury and pollution are also noted as potentially modifiable risk factors [25].

Further, an array of socio-demographic factors is associated with an increased risk of dementia including living in areas of socio-economic deprivation [26; 27] and areas with low educational attainment [28]. Loneliness is linked to an increased risk of dementia [29] and social isolation has been shown to increase the risk of dementia by 50 percent [30]. The impact of both of these factors has been exacerbated by the COVID-19 pandemic [31]. The consensus among international experts at the G8 Dementia Summit in 2013 was that increasing physical activity and fruit and vegetable consumption, controlling weight, diabetes and high blood pressure, smoking cessation and avoiding excessive alcohol intake could prevent a substantial proportion of dementia cases [32].

Prevalence and cost of dementia

The current estimates of dementia prevalence in the UK indicate that around 7.1 per cent, representing 1 in every 14 of the population aged 65 years and older are diagnosed with dementia [33]. Similarly, in North America, 10 per cent of individuals aged 65 and over receive a dementia diagnosis [34]. However, a substantial number of cases are not diagnosed, leading to difficulties in estimating true prevalence. There is a pressing need for high-quality data from nationally representative samples of older men and women, hence the need for the HCAP study [34]. Reliable national data on incidence and prevalence of dementia and cognitive impairment are vital for service planning, the prediction of future needs, estimating the costs of dementia care and understanding the impact of these conditions on individuals and their families. There is also a strong research need for robust estimates of MCI and dementia in order to facilitate studies of the aetiology and consequences of these conditions.

For peer review only

Although the number of people with dementia is projected to increase throughout the world because of a well-documented demographic shift towards rising numbers of older adults [35], estimates of future levels are complicated by the assumptions of the underlying methods used to derive them. There is evidence from both England and the US that the incidence of dementia has declined over the past decades [36] but it is rising exponentially in low and middle income countries [37].

The marked increase in numbers of individuals expected to have dementia has important economic implications. With an estimate of 13 trillion US dollars annually attributed to the condition, an increase in prevalence will have significant economic impact globally [38]. The corresponding economic cost to the UK of caring for people with dementia is estimated to grow from £34.7 billion (2019) to £94.1 billion by 2040 [39]. As the population ages, a greater proportion of people in the UK and elsewhere will be living with dementia, and more of the country's resources will be required to care for them.

Policy context and relevance

The importance of dementia has been highlighted with the publication of a national dementia strategy for England [40] and Northern Ireland [41], and in the ex-Prime Minister Cameron's 'Challenge on Dementia' in 2012 [42]. The latter emphasised the central role of three issues: raising awareness of dementia and reducing social isolation that people with dementia feel; improving the quality of care; and increasing research. Good quality research generates awareness, shapes policy and encourages service development. However, there is an ongoing need for robust estimates of dementia. Evidence based on data from the NHS Quality and Outcome Framework suggests that 56 per cent of dementia cases in England are not formally diagnosed, with wide variations between areas of the country [43]. Therefore, it is hard to reach a clear consensus about the true dementia prevalence in the UK and elsewhere.

Methods and Analysis

Study Aims and Objectives

The aim of the NICOLA-HCAP study is to collect detailed cognitive data in a subset (n=1000) of the NICOLA cohort using an enhanced harmonised protocol assessment, with measures that are currently administered in the US-based HRS [2]. This will enable estimation of the prevalence of cognitive impairment and dementia among older adults and comparisons of the rates of dementia prevalence in the US and Ireland. The TILDA study is conducting their HCAP study concurrently with NICOLA-HCAP, thus enabling the comparison of rates across Ireland. The findings will increase understanding of the personal, social and economic burden of dementia. The study has five main objectives.

For peer review only - http://bmjopen.bmj.com/site/about/guidelines.xhtml

Objective 1: Collect in-depth interview assessments.

The collection of in-depth interview assessments involves the administration of standardised NICOLA-HCAP interviews, both with respondents and their family member or friend ('informant'). The protocol was developed by the HRS HCAP for the international network [44]. The respondent's cognitive ability is measured using a computer-assisted personal interview (CAPI). A family member or friend is asked to complete an additional interview of validated measures to provide information on respondent's prior and current cognitive and physical functioning and whether the individual requires help with daily activities, two core clinical criteria required for dementia diagnosis [44].

Objective 2: Harmonise scoring and investigate the prevalence of dementia and cognitive impairment.

Harmonisation of the scoring of the NICOLA-HCAP data to that of HCAP studies conducted in Ireland (TILDA), the United States (HRS) and England (ELSA) will take place. As the work aims to establish research diagnoses of probable presence or absence of dementia and cognitive impairment, algorithms will be developed to ascertain the probable presence or absence of dementia and cognitive impairment in this sub-sample of NICOLA, in collaboration with colleagues from TILDA, HRS and ELSA. Establishing clear assessments of dementia or MCI prevalence rates in the study will allow the extrapolation of dementia rates from this sub-sample to the rest of the NICOLA population in general. As a result, this information will provide the first estimates of dementia and cognitive impairment from a nationally representative sample in Northern Ireland.

Objective 3: Explore the validity of dementia estimates.

The work will involve the construction of estimates of dementia prevalence for the national populations of Northern Ireland and Ireland harmonised to estimates for the United States and England, having first established the representativeness of the national cohorts, and the development of population weights to account for non-random participation and institutionalisation (care homes). Relationships between the harmonised HCAP data and the more limited cognitive and functioning measures in the core Irish studies will be analysed to establish comparable research diagnoses in the full samples to further expand the data available for epidemiologic study, and identify measures that might be added to core studies to improve cognitive assessment going forward.

Objective 4: Investigate the risk factors for dementia and cognitive impairment

Preliminary analyses will be conducted to: a) Investigate potential causal pathways from cardiovascular instability, cerebral microvascular disease and sensory impairment to dementia using

For peer review only

objective measures from the TILDA and NICOLA health assessments, including neuroimaging, near-infrared spectroscopy, hearing, vision, retinal photography, and multi-sensory integration technologies and using polygenic scores to capture genetic differences; b) Use cross-country analyses to investigate how stress and long-term exposure to conflict in a unique cohort of older adults affects cognitive aging and physiological ageing and methylation / epigenetic patterns in known risk loci related to traumatic stress; c) Establish potentially modifiable risk factors for primary and secondary prevention of dementia.

Objective 5: Provide reliable data to researchers.

Data from NICOLA-HCAP will be provided to the research community to complement existing public data resources from these studies and support epidemiological research being conducted elsewhere.

Study Design

The NICOLA-HCAP assessment is comprised of two main parts: a face-to-face interview with the participant, and a shorter interview, with a family member or friend, nominated by the participant (the ‘informant’).

Participant interview

The participant interview is administered face-to-face using a computer assisted personal interview (CAPI) formatted using Epi Info software package [45]. The interview takes place in the participant’s home, however, the option of attending the Centre for Public Health at Queen’s University Belfast or the Clinical Translational Research and Innovation Centre (C-TRIC) based in Derry/Londonderry is offered to those who prefer not to have a researcher visit them at home. The interview is approximately one hour in duration and consists of nineteen different measures of memory, language, attention and other aspects of cognitive ability. The Centre for Epidemiologic Studies Depression Scale (CES-D) is administered as an assessment of mental wellbeing to adjust for the impact of depressive symptoms on cognitive test performance (see Table 1). The majority of measures are completed verbally by the participant, with the researcher recording answers on the laptop, while some other measures are completed by the participant using paper and pencil (due to nature of the test and licencing restrictions). Interviews with participants are audio recorded (only with participants’ consent) on the laptop for internal quality control processes. All participants are offered a gift voucher (20 GBP) in appreciation of their participation in the study.

For peer review only - http://bmjopen.bmj.com/site/about/guidelines.xhtml

Table 1: List of outcome measures from participant CAPI interview [44]

OUTCOME MEASURE	COGNITIVE DOMAIN
Mini-Mental State Examination (MMSE) [49]	Global cognitive status
HRS Telephone Interview for Cognitive Status (HRS-TICS)	Global cognitive status
CERAD Word List Learning and Recall – Immediate	Episodic memory (immediate)
Retrieval Fluency (Animal naming)	Language/fluency
Letter Cancellation	Attention/processing speed
Backward Count	Attention/processing speed
10/66 Respondent	Global cognitive status
CERAD Word List Recall - Delayed	Episodic memory (delayed)
Story recall – Immediate	Episodic memory (immediate)
CERAD Word List - Recognition	Episodic memory (recognition)
CERAD Constructional Praxis – Immediate	Visuospatial ability
Symbol-Digit Modalities Test	Attention/processing speed
CERAD Constructional Praxis – Delayed	Episodic memory (delayed)
Story recall – Delayed	Episodic memory (delayed)
Story recall – Recognition	Episodic memory (recognition)
HRS Number Series	Executive function
Raven's Standard Progressive Matrices	Abstract reasoning
Trail Making Test (Part A & B)	Executive function/ attention/processing speed
CES-D Depressive Symptoms	Assessment of presence and level of depression

Note. Tests listed in order of administration
CES-D, Centre for Epidemiological Studies Depression Scale
CERAD, Consortium to establish a registry for Alzheimer's disease

The NICOLA-HCAP interview is replicated on the HRS study and contains the same measures administered in the US. These measures are standardised measures for the identification and diagnosis of cognitive impairment and dementia administered across the globe, including in Memory Clinics in Northern Ireland. The HCAP assessment was adapted from the ADAMS study [46] which was originally designed to provide the first national estimates of dementia in the US. The ADAMS study included a three hour cognitive assessment. HCAP was designed to be less intensive (i.e. one hour) than the ADAMS study in order to reduce burden and fatigue for participants. Care is also taken with participants who were physically frail or facing cognitive issues to ensure that they understood the content and purpose of the study, its voluntary nature and that the burden of participating in the study was not too great for them. The research nurses were also experienced in working with older people including those with cognitive decline.

Family or friend (informant) interview

For peer review only - http://bmjopen.bmj.com/site/about/guidelines.xhtml

During the home interview, the participant is asked to nominate a family member or friend (the 'informant') who has known them for some years and whom they think would be willing to answer some questions about the participant and their daily activities. The family or friend interview includes questions relating to changes in the participant's cognitive abilities over time, and activities the participant carries out inside or outside the home (see Table 2). As with the participant interview, the family or friend interview is also administered via CAPI in Epi Info [45] and is audio recorded for quality control purposes. The interview lasts approximately twenty minutes and is administered at the participant's home if the family member or friend is present at the time of the participant interview. If they are not present, the family member or friend is contacted by telephone and the interview is completed over the phone at a time convenient to them. Alternatively, the family member or friend can request a paper version of the questionnaire to be posted to their home or they can complete the questionnaire online (based on Qualtrics user platform [47]) whereby a link to the survey is emailed or texted to their mobile phone. The participant can request someone to be present during their interview for support however it is preferred the informant interview is completed one-to-one with the research nurse (or online) to minimise bias based on the presence of the participant.

Table 2: List of outcome measures from informant CAPI interview [44]

OUTCOME MEASURE	DESCRIPTION
Family or friend demographics	Demographic profile of informant
JORM IQCODE	Assessment of cognitive decline
Blessed dementia rating scale - Part 2	Assessment of ability to do basic self-care activities
HRS Activities questionnaire	Assessment of participant activity engagement
CSI-D – Cognitive Activities Questionnaire	Assessment of participant's cognitive activity engagement and ability
10/66 Informant Questionnaire	Assessment of ability to do daily activities
Blessed dementia rating scale - Part 1	Assessment of additional activities and mental ability

Note. Tests listed in order of administration.
IQCODE, informant questionnaire on cognitive decline in the elderly
CSI-D, Community Screening Instrument for Dementia

Sample

The NICOLA-HCAP sample is drawn from core members of the NICOLA cohort. The aim is to recruit 1000 participants who took part in the NICOLA Wave 2 CAPI (end date December 2019), aged 65 years or over at the start of HCAP fieldwork. The data from this sample is being combined with the same HCAP data from our sister study TILDA, based in the Republic of Ireland and the ELSA study in England. The combined sample is equivalent to the sample size of the HRS HCAP sample.

For peer review only - http://bmjopen.bmj.com/site/about/guidelines.xhtml

Both participant and informant interviews are conducted with participants who are residing in private residential accommodation in Northern Ireland. Those who were in institutionalised care i.e. residential homes or nursing homes were excluded from participating in NICOLA and therefore will not be included in NICOLA-HCAP. Individuals are randomly selected and the sample is stratified to include participants who live alone (single household) and participants who do not live alone (multiple household). Participants in multiple households who are initially ineligible but become eligible later (typically spouses who turn 65 after the onset of the study) are also able to take part. An 80 per cent response rate is expected to the NICOLA-HCAP, which implies a selection rate of about 1 in 3 to achieve the target of 1,000 participants. Individuals will be excluded from the study if they are unable to complete the written tasks included in the NICOLA-HCAP assessment (due to reasons such as sensory impairment, e.g. hearing loss, visual impairment) or if they are not fluent in English. As the proportion of non-English speakers was low in the NICOLA over 65 cohort, the HCAP assessment was not translated to other languages. However, other studies in the network have translated the assessment and therefore it is possible for further NICOLA - HCAP waves.

A protocol was developed for those individuals that were deemed to not have capacity to consent. In the main waves of NICOLA, if a participant is thought to not be mentally capable of giving informed consent (if, for example, they are too confused to be able to fully understand what they are consenting to), a consultee can be approached to decide whether or not the participant should be included in that wave of NICOLA. For the HCAP sub-study, if it is deemed that the participant does not have the capacity to consent, they can nominate a friend/relative or carer to act as a consultee. The consultee will be asked by the researcher whether they believed that, were the participant able to consent, they would agree to take part in the HCAP sub-study within NICOLA. The consultee will then sign a Consultee Declaration Form confirming that in their opinion the participant would or would not want to take part in the study

Informants are nominated by participants and can be a family member, friend or carer. The researcher discusses the nomination with the participant once they have agreed to take part in the interview, and recommends that the nominated person is someone who knows the participant well and sees them frequently enough to answer questions about their daily life, as well as being someone who has known them for a number of years. The recommendation is that the nominated person is a family member or friend rather than someone who is employed to be with the participant, such as a carer, however, if a family member or friend is not an option and the employed person has known the participant for long enough, they can fulfil this role. Inability to nominate an informant is not an exclusion criteria;

For peer review only - http://bmjopen.bmj.com/site/about/guidelines.xhtml

should the participant refuse to nominate or the informant refuse to take part, NICOLA-HCAP will follow the HRS protocol for the imputation of missing data.

Proposed statistical methods

We will develop algorithms to ascertain the probable presence of absence of dementia and cognitive impairment in this sub-sample of NICOLA, in collaboration with colleagues from TILDA, HRS and the ELSA. Establishing clear estimates of dementia or mild cognitive impairment prevalence rates in the study will allow the extrapolation of dementia rates from this sub-sample to the rest of the NICOLA population in general. As a result, we will be able to provide the first estimates of dementia and cognitive impairment from a nationally representative sample in Northern Ireland.

We will harmonise the scoring of the Northern Irish HCAP data to that of HCAP studies conducted in Ireland (TILDA), the US (HRS) and England (ELSA) and establish research diagnoses of probable presence or absence of dementia and cognitive impairment in these subsamples of NICOLA using algorithms developed in collaboration with HRS and ELSA based on harmonised measures. We will construct estimates of dementia prevalence for the national populations of Ireland and Northern Ireland harmonized to estimates for US and England, having first established the representativeness of the national cohorts, and develop population weights to account for non-random participation and institutionalisation (care homes). Sample weights will be applied as per the HRS protocol adjusting for differential sampling weights by race/ethnicity, age, cognitive ability and for differential participation by demographic factors such as education, socio-economic status etc.

The use of the one-hour battery of identical tests, twenty minutes of informant interview, and similar methods of administration, provides a strong foundation for harmonisation. Subtle differences in scoring can produce disparities, so there will be extensive comparison across studies using audio recordings, participant drawings, and other records to ensure comparability. Led by HRS, statistical procedures such as tests for factor invariance will be used to determine if there are any specific inconsistencies in tests between countries. Similar analyses will be carried out with the informant reports.

We will analyse the relationships between the harmonized HCAP data and the more limited cognitive and functioning measures in the core Irish studies to establish comparable research diagnoses in the full samples to further expand the data available for epidemiologic study, and identify measures that might be added to core studies to improve cognitive assessment going forward.

For peer review only - http://bmjopen.bmj.com/site/about/guidelines.xhtml

We will conduct preliminary analyses to: a) Investigate potential causal pathways from cardiovascular instability, cerebral microvascular disease and sensory impairment to dementia using objective measures in TILDA and NICOLA health assessments, including neuroimaging, near-infrared spectroscopy, hearing, vision, retinal photography, and multi-sensory integration technologies and using polygenic scores to capture genetic differences b) Use cross-country analyses to investigate how stress and long-term exposure to conflict in a unique cohort of older adults affects cognitive aging and physiological ageing and methylation / epigenetic patterns in known risk loci related to traumatic stress c) Establish potentially modifiable risk factors for primary and secondary prevention of dementia.

Staff Training

Fieldwork staff working on the NICOLA-HCAP study includes both Queen's University research staff and dementia trained clinical research nurses from the Northern Ireland Clinical Research Network (NICRN). All staff received face-to-face training delivered in two phases. Phase one focused on study orientation and the development of the Standard Operating Procedures (SOP). In brief, sessions with fieldwork staff included an introduction to the research aims and objectives; study design; staffing roles; recruitment procedures and pre-visit protocols; consent procedures and capacity to consent protocols; set up of Epi Info software [45] (for data capture – participant CAPI and informant CAPI) and the upload of data to secure servers. Phase two focused on the administration of cognitive tests and informant measures. The Survey Research Operations (SRO) team based in the Survey Research Centre in the University of Michigan developed training videos and scripts for in-house training. Fieldwork staff met in the first instance as a full team to review the videos and practice administration using the CAPI software. This was followed by practice administration using a buddy system. This involved fieldwork staff working in pairs, with one member of staff adopting the role of the participant and the other the interviewer (and vice versa). As training for TILDA-HCAP was running consecutively with NICOLA-HCAP, joint TILDA-NICOLA meetings were held with the SRO and HRS team to address any queries relating to test administration and scoring. The HRS team subsequently developed a scoring protocol to ensure consistency across studies.

Patient and Public Involvement (PPI)

The pilot study included participants from the NICOLA Healthy Aging Research Advisory Group. This group consisted of both NICOLA participants and individuals from AgeNI, a local NI based charitable

For peer review only - http://bmjopen.bmj.com/site/about/guidelines.xhtml

organisation. These participants gave feedback during the pilot phase in relation to the length of the assessment, content and overall process. Further detail is noted below.

NICOLA-HCAP Dress Rehearsal

The pilot dress rehearsal mirrored the ELSA-HCAP dress rehearsal [48] and was used to inform the mainstage survey. The main aim of the pilot was to implement the study as per the NICOLA-HCAP study protocol and to identify any areas that needed to be revised or improved in relation to the study design, questionnaire format and content, data collection, data upload processes and materials prior to the main phase and the SOP could be amended accordingly. The pilot dress-rehearsal was conducted between the 6th January 2022 and 25th January 2022.

Pilot Design

The dress rehearsal aimed to recruit 15 participants and 15 family or friend members to take part in the respective interviews. Following each interview, the research nurses respectively completed an interview feedback form that included sections for feedback on both fieldwork procedures and in-house processes. In brief, the fieldwork feedback included information on personal protective equipment (PPE) requirements and any perceived impact on study administration; participant information and consent processes; CAPI and paper administrations; and participant experience. Feedback on the in-house processes centred on the administrative procedures; pre-visit protocols; use of secure servers and the efficiency of the process required to carry out data uploads of participant and family or friend interviews; data storage and any additionally required processes to support the smooth delivery of the project.

Pilot Sample

A total of 14 participant interviews and 14 informant interviews were achieved within the timeframe for the pilot study. The pilot dress rehearsal sample was selected from the NICOLA Healthy Ageing Research Advisory panel which included both NICOLA participants (n=5) and volunteers from AgeNI [49] (n=9) along with their respective family member or friend. Members of the Advisory Panel understood that their role was to test the design, content and materials of the proposed NICOLA-HCAP interview. Ethical approval for the pilot was obtained from the Faculty of Medicine, Health and Life Sciences Research Ethics Committee, Queen's University Belfast.

Pilot Revisions

For peer review only - http://bmjopen.bmj.com/site/about/guidelines.xhtml

Feedback obtained from fieldwork staff informed a number of study revisions or improvements that were integrated into the SOP and implemented for the main study. The pilot identified the following key areas for improvement.

Area one: Pre-Visit Documentation. Fieldwork staff reflected on the complexities of test administration and the transition between the CAPI and paper versions of tests. Pre-visit protocols were revised to include preparation processes (i.e. folders with test papers organised in order of test administration and participant identification numbers pre-added to documentation). Staff also preferred a manual timer for tests rather than the built-in CAPI version.

Area two: Test Administration. Queries were raised in relation to the logistics of some test administration. Fieldwork staff also questioned how tests were scored and entered into the Epi Info software. All queries were resolved with the HRS and TILDA teams.

Area three: Study Administration Processes. Additional administration databases were set up to ease the flow of recruitment and home visit bookings. In addition, the SOP was amended to update in-house filing and the secure storage of data.

Ethics and Dissemination

The main study received ethical approval from the Faculty of Medicine, Health and Life Sciences Research Ethics Committee, Queen's University Belfast [50]. Participants and informants provided written informed consent prior to participation in the study. The NICOLA-HCAP main study commenced on 7th February 2022 with fieldwork due to complete in June 2023. The data from NICOLA-HCAP is currently held in the data repository within the Centre for Public Health at Queen's University Belfast and post study completion, all NICOLA-HCAP data will be provided to the research community via data repositories such as the Dementias Platform UK (DPUK) [51] and Gateway to Global Aging to complement existing public data resources and support epidemiologic research by others. NICOLA Wave 1 and Wave 2 data are also maintained and stored at a data repository within the Centre for Public Health, Queen's University Belfast. Information on how to access NICOLA data and details regarding the data that is currently available in NICOLA can be found on the study website [52]. Findings from this study will also be disseminated through peer reviewed publications and via presentations at international conferences.

Acknowledgements

For peer review only - http://bmjopen.bmj.com/site/about/guidelines.xhtml

The authors would like to acknowledge the participation of members from the NICOLA Healthy Ageing Research Advisory panel in the NICOLA-HCAP pilot study. Your participation and feedback on the process was invaluable to the study. The authors are also grateful to the participants from the NICOLA study, and the wider NICOLA team for their continued support and assistance and would like to thank the research nurses, clerical staff and computer technicians for their time, dedication, professionalism and contribution to the NICOLA-HCAP study. The authors alone are responsible for the interpretation of the data and any views or opinions presented are solely those of the authors and do not necessarily represent those of the NICOLA study team.

Author contributions

LO, CN, CM, MM, FK, DW, BM have made substantial contributions to the conception or design of the work; or the acquisition, analysis, or interpretation of data for the work. LO, CN, CM, MM, FK, DW, BM were involved in drafting the work or revising it critically for important intellectual content. LO, CN, CM, MM, FK, DW, BM gave final approval of the version to be published and agreed to be accountable for all aspects of the work in ensuring that questions related to the accuracy or integrity of any part of the work are appropriately investigated and resolved.

Funding

The Northern Ireland Cohort for the Longitudinal Study of Ageing Harmonised Cognitive Assessment Protocol (NICOLA-HCAP) is funded by the National Institute on Aging (NIA; Grant number (GN) SUBK00010180). The NICOLA study (Waves 1 and 2) is funded by the Atlantic Philanthropies (GN – 18107), the Economic and Social Research Council (GN -ES/L0084559/1); the UKCRC Centre for Excellence for Public Health Northern Ireland (GN- N/A); Northern Ireland Health and Social Care Research and Development Division of the Public Health Agency (GN - STL/4717/12); the Wellcome Trust/Wolfson Foundation (GN- N/A), the Centre for Ageing Research and Development Ireland (GN- N/A), Queen's University Belfast (GN- N/A) and the Office of the First Minister and Deputy First Minister Northern Ireland (GN- N/A).

Competing Interests.

None declared.

For peer review only - http://bmjopen.bmj.com/site/about/guidelines.xhtml

References

[1] Neville C*, Burns F*, Cruise S, Scott A, O'Reilly D, Kee F, Young I. Cohort Profile: the Northern Ireland Cohort for the Longitudinal Study of Ageing (NICOLA). International Journal of Epidemiology. 2023. Doi: doi: 10.1093/ije/dyad026. Epub ahead of print. PMID: 37011634.

[2] Health and Retirement Study (HRS). https://hrsonline.isr.umich.edu/ (14th March 2023, date last accessed).

[3] Irish Longitudinal Study on Ageing (TILDA). https://tilda.tcd.ie/ (14th March 2023, date last accessed).

[4] English Longitudinal Study of Ageing (ELSA). https://www.elsa-project.ac.uk/ (14th March 2023, date last accessed).

[5] Cruise S, Kee F (Eds). Early key findings from a study of older people in Northern Ireland: The NICOLA Study. Belfast, UK: Queen's University Belfast, 2017. https://www.qub.ac.uk/sites/NICOLA/ReportsandNewsletters/. (14th March 2023, date last accessed)

[6] World Health Organisation. Dementia. https://www.who.int/news-room/fact-sheets/detail/dementia (14th March 2023, date last accessed).

[7] Office for National Statistics. https://www.ons.gov.uk/peoplepopulationandcommunity/healthandsocialcare/causesofdeath/articles/leadingcausesofdeathuk/2001to2018 (14th March 2023, date last accessed).

[8] National Institute on Aging. What is Dementia? Symptoms, Types and Diagnosis. https://www.nia.nih.gov/health/what-is-dementia (14th March 2023, date last accessed).

[9] White S, Griffiths R, Baxter M, Beanland T, Cross J, Dhesi J, Docherty A.B, Foo I, Jolly G, Jones J, Moppett I.K, Plunkett E, Sachdev K. Guidelines for the peri-operative care of people with dementia. Anaesthesia. 2019; 74: 271-273.

[10] World Health Organisation. Dementia.https://www.who.int/news-room/fact-sheets/detail/dementia (14th March 2023, date last accessed).

[11] Bassett S.S, & Folstein M.F. Cognitive impairment and functional disability in the absence of psychiatric diagnosis. Psychol Med. 1991; 21(1): 77-84.

[12] Alzheimer's Society. https://www.alzheimers.org.uk/about-dementia/types-dementia/mild-cognitive-impairment-mci (14th March 2023, date last accessed).

[13] Mayo Clinic. https://www.mayoclinic.org/diseases-conditions/mild-cognitive-impairment/symptoms-causes/syc-20354578 (14th March 2023, date last accessed).

For peer review only - http://bmjopen.bmj.com/site/about/guidelines.xhtml

[14] Petersen C.P, et al. Cognitive Impairment: Clinical Characterization and Outcome. Arch Neurol. 1999; 56: 303-308.

[15] Alzheimer's Disease Facts and Figures https://www.cdc.gov/aging/pdf/cognitive_impairment/cogimp_poilicy_final.pdf (01 March 2023, date last accessed).

[16] World Health Organisation. Dementia. https://www.who.int/news-room/fact-sheets/detail/dementia (14th March 2023, date last accessed).

[17] Yan S, Wennning F, Wang C, Mao J, Bing L, Zou L, Lv C. Association between sedentary behaviour and the risk of dementia: A systematic review and meta-analysis. Transitional Psychiatry. 2020; 10; 112. https://doi.org/10.1038/s41398-020-0799-5.

[18] Choi D, Choi S, Park S M. Effect of smoking cessation on the risk of dementia: a longitudinal study. Annals of Clinical and Translational Neurology. 2018; 5 (10); 1192-1199. doi: 10.1002/acn3.633.

[19] Wiegmann C, Mick I, Brandl E J, Heinz A, Gutwinski S. Alcohol and Dementia - What is the link? A systematic review. Neuropsychiatric Disease and Treatment. 2020; 16; 87-99.

[20] Ma Y, Ajnakina O, Steptoe A, Cadar D. Higher risk of Dementia in English older individuals who are overweight or obese. International Journal of Epidemiology. 2020; 1353-1365. doi: 10.1093/ije/dyaa099.

[21] de Crom T O E, Mooldijk S S, Ikram M K, Ikram M A, Voortman T. MIND diet and the risk of dementia: a population-based study. Alzheimer's Research and Therapy. 2022 14; 8. https://doi.org/10.1186/s13195-022-00957-1.

[22] Varma V R, Wang Y, An Y, Varma S, Bigel M, Doshi J, et al. Bile acid synthesis, modulation and dementia: A metabolomic, transcriptomic, and pharmacoepidemiologic study. PLOS Medicine. 2021; 18 (5). doi:10.1371/journal.pmed.1003615.

[23] Crane PK, Walker R, Hubbard, R A, Li G, Nathan D M, et al. Glucose levels and risk of dementia. N Engl J Med. 2013; 369; 540. DOI: 10.1056/NEJMoa1215740

[24] Lee C J, Lee Ji-L, Han K, Kim D H, Cho H, Kim K J, et al. Blood pressure levels and risks of dementia: a Nationwide study of 4.5 million people. Hypertension. 2022; 79 (1); 218-229.

[25] Livingstone G, Huntley J, Sommerlad A, Ames D, Ballard C, Banerjee S, et al. Dementia prevention, intervention and care: 2020 report of the Lancet Commission. The Lancet Commissions. 2020; 396 (10248); 413-446.

[26] Jitlal M, Amirthalingam G N K, Karania T, Parry E, Neligan A, et al. The Influence of Socioeconomic Deprivation on Dementia Mortality, Age at Death, and Quality of Diagnosis: A Nationwide Death Records Study in England and Wales 2001–2017. J Alzheimers Dis. 2021; 81 (1) 321 – 328. doi: 10.3233/JAD-210089.

For peer review only

[27] McMichael A J, Zafeiridi E, Passmore P, Cunningham E L, McGuinness B. Factors associated with mortality including nursing home transitions: A retrospective analysis of 25,418 people prescribed anti-dementia drugs in Northern Ireland. J Alzheimer's Disease. 2020; 73; 1233-1242. DOI 10.3233/JAD-190751.

[28] Takasugi T, Tsuji T, Hanazato M, Miyaguni, Y, et al. Community-level educational attainment and dementia: a 6-year longitudinal multilevel study in Japan. BMC Geriatrics. 2021; 21:661. https://doi.org/10.1186/s12877-021-02615-x.

[29] Lara E, Martín-María N, De la Torre-Luque A, Koyanagi A, Vancampfort D, Izquierdo A, et al. Does loneliness contribute to mild cognitive impairment and dementia? a systematic review and meta-analysis of longitudinal studies. *Ageing Res. Rev.* 52, 7–16. doi: 10.1016/j.arr.2019.03.002

[30] National Academies of Sciences, Engineering, and Medicine. *Social Isolation and Loneliness in Older Adults: Opportunities for the Health Care System*. 2020. Washington, DC: The National Academies Press**.**

[31] Belfast City Council. Age Friendly Belfast Plan 2022-2026. https://yoursay.belfastcity.gov.uk/age-friendly-plan-2022-26. 2022. (14th March 2023, date last accessed).

[32] Department of Health and Social Care. https://www.gov.uk/government/publications/g8-dementia-summit-global-action-against-dementia/g8-dementia-summit-global-action-against-dementia-11-december-2013 (14th March 2023, date last accessed)**.**

[33] Alzheimer's Society https://www.alzheimers.org.uk/about-us/policy-and-influencing/dementia-uk-report (14th March 2023, date last accessed).

[34] Manly, J.J., Jones, R.N., Langa, K.M., Ryan, L.H., Levine, D.A., McCammon, R., Heeringa, S.G. and Weir, D., 2022. Estimating the prevalence of dementia and mild cognitive impairment in the US: the 2016 Health and Retirement Study harmonized cognitive assessment protocol project. *JAMA neurology*, *79*(12), pp.1242-1249.

[35] World Health Organisation. https://www.who.int/news-room/fact-sheets/detail/ageing-and-health (14th March 2023, date last accessed).

[36] Matthews F.E. et al. A two decade dementia incidence comparison from the Cognitive Function and Ageing Studies 1 and 11. Nature Communications. 2016; 7: 11398. https://doi.org/10.1038/ncomms11398

[37] Pedroza P. et al. Global and regional spending on dementia care from 2000-2019 and expected future health spending scenarios from 2020-2050: An economic modelling exercise. The Lancet. 2022; 45: 101337. DOI: https://doi.org/10.1016/j.eclinm.2022.101337.

For peer review only

[38] Livingstone G, Huntley J, Sommerlad A, Ames D, Ballard C, Banerjee S, et al. Dementia prevention, intervention and care: 2020 report of the Lancet Commission. The Lancet Commissions. 2020; 396 (10248); 413-446.

[39] Alzheimer's Society. https://www.alzheimers.org.uk/about-us/policy-and-influencing/dementia-scale-impact-numbers (14th March 2023, date last accessed).

[40] Department of Health and Social Care. https://www.gov.uk/government/publications/living-well-with-dementia-a-national-dementia-strategy. (14th March 2023, date last accessed).

[41] Department of Health. https://www.health-ni.gov.uk/publications/improving-dementia-services-northern-ireland-regional-strategy (14th March 2023, date last accessed).

[42] Department of Health and Social Care. https://www.gov.uk/government/publications/prime-ministers-challenge-on-dementia (14th March 2023, date last accessed).

[43] NHS.https://digital.nhs.uk/data-and-information/publications/statistical/nhs-outcomes-framework (14th March 2023, date last accessed).

[44] Langa KM, Ryan LH, McCammon R, Jones RN, Manly JJ, Levine DA, Sonnega A, Farron M & Weir D. The Health and Retirement Study Harmonized Cognitive Assessment Protocol (HCAP) Project: Study Design and Methods. Neuroepidemiology. 2021; 54(1):64-74. DOI: 10.1159/000503004.

[45] Centres for Disease Control and Prevention. https://www.cdc.gov/epiinfo/index.html (14th March 2023, date last accessed).

[46] Langa KM, Plassman BL, Wallace RB, Herzog AR, Heeringa SG, Ofstedal MB, Burke JR, Fisher GG, Fultz NH, Hurd NH, Potter GG, Rodgers WL, Steffens DC, Weir DR & Willis RJ. The Aging, Demographics, and Memory Study: study design and methods. Neuroepidemiology 2005; 25(4): 181-91. DOI: 10.1159/000087448.

[47] Qualtrics XM. https://www.qualtrics.com/uk/ (14th March 2023, date last accessed).

[48] Cadar D, Abell J, Matthews FE, Brayne C, David Batty G, Llewellyn DJ, et al. Cohort Profile Update: The Harmonised Cognitive Assessment Protocol Sub-study of the English Longitudinal Study of Ageing (ELSA-HCAP). Int J Epidemiol. 2021;50(3):725-6i. DOI:10.1093/ije/dyaa227 pmcid:PMC8271185

[49] AgeNI. https://www.ageni.org/ (14th March 2023, date last accessed).

[50] Faculty of Medicine, Health and Life Sciences Research Ethics Committee, Queen's University Belfast https://www.qub.ac.uk/Research/Governance-ethics-and-integrity/Ethics/FacultyResearchEthicsCommittees/ (14th March 2023, date last accessed).

[51] Dementias Platform UK. https://www.dementiasplatform.uk/. (14th March 2023, date last accessed).

[52] NICOLA. https://www.qub.ac.uk/sites/NICOLA/Informationforresearchers/ (14th March 2023, date last accessed).

For peer review only - http://bmjopen.bmj.com/site/about/guidelines.xhtml

STROBE Statement—checklist of items that should be included in reports of observational studies

	Item No.	Recommendation	Page No.	Relevant text from manuscript
**Title and abstract**	1	(*a*) Indicate the study's design with a commonly used term in the title or the abstract	1	
		(*b*) Provide in the abstract an informative and balanced summary of what was done and what was found	2	
**Introduction**				
Background/rationale	2	Explain the scientific background and rationale for the investigation being reported	3	
Objectives	3	State specific objectives, including any prespecified hypotheses	6	
**Methods**				
Study design	4	Present key elements of study design early in the paper	8	
Setting	5	Describe the setting, locations, and relevant dates, including periods of recruitment, exposure, follow-up, and data collection	10	
Participants	6	(*a*) *Cohort study*—Give the eligibility criteria, and the sources and methods of selection of participants. Describe methods of follow-up<br>*Case-control study*—Give the eligibility criteria, and the sources and methods of case ascertainment and control selection. Give the rationale for the choice of cases and controls<br>*Cross-sectional study*—Give the eligibility criteria, and the sources and methods of selection of participants	11	
		(*b*) *Cohort study*—For matched studies, give matching criteria and number of exposed and unexposed<br>*Case-control study*—For matched studies, give matching criteria and the number of controls per case	N/A	
Variables	7	Clearly define all outcomes, exposures, predictors, potential confounders, and effect modifiers. Give diagnostic criteria, if applicable	9-10	
Data sources/ measurement	8*	For each variable of interest, give sources of data and details of methods of assessment (measurement). Describe comparability of assessment methods if there is more than one group	*9-10*	
Bias	9	Describe any efforts to address potential sources of bias	N/A	
Study size	10	Explain how the study size was arrived at	10	

Continued on next page

For peer review only - http://bmjopen.bmj.com/site/about/guidelines.xhtml

For peer review only

Quantitative variables	11	Explain how quantitative variables were handled in the analyses. If applicable, describe which groupings were chosen and why	N/A
Statistical methods	12	(*a*) Describe all statistical methods, including those used to control for confounding	12
		(*b*) Describe any methods used to examine subgroups and interactions	12-13
		(*c*) Explain how missing data were addressed	12
		(*d*) *Cohort study*—If applicable, explain how loss to follow-up was addressed<br>*Case-control study*—If applicable, explain how matching of cases and controls was addressed<br>*Cross-sectional study*—If applicable, describe analytical methods taking account of sampling strategy	12
		(*e*) Describe any sensitivity analyses	N/A
**Results**			
Participants	13*	(a) Report numbers of individuals at each stage of study—eg numbers potentially eligible, examined for eligibility, confirmed eligible, included in the study, completing follow-up, and analysed	N/A
		(b) Give reasons for non-participation at each stage	N/A
		(c) Consider use of a flow diagram	N/A
Descriptive data	14*	(a) Give characteristics of study participants (eg demographic, clinical, social) and information on exposures and potential confounders	N/A
		(b) Indicate number of participants with missing data for each variable of interest	N/A
		(c) *Cohort study*—Summarise follow-up time (eg, average and total amount)	N/A
Outcome data	15*	*Cohort study*—Report numbers of outcome events or summary measures over time	*N/A*
		Case-control study—Report numbers in each exposure category, or summary measures of exposure	*N/A*
		Cross-sectional study—Report numbers of outcome events or summary measures	*N/A*
Main results	16	(*a*) Give unadjusted estimates and, if applicable, confounder-adjusted estimates and their precision (eg, 95% confidence interval). Make clear which confounders were adjusted for and why they were included	N/A
		(*b*) Report category boundaries when continuous variables were categorized	N/A
		(*c*) If relevant, consider translating estimates of relative risk into absolute risk for a meaningful time period	N/A

Continued on next page

For peer review only - http://bmjopen.bmj.com/site/about/guidelines.xhtml

For peer review only

Other analyses	17	Report other analyses done—eg analyses of subgroups and interactions, and sensitivity analyses	N/A
**Discussion**			
Key results	18	Summarise key results with reference to study objectives	N/A
Limitations	19	Discuss limitations of the study, taking into account sources of potential bias or imprecision. Discuss both direction and magnitude of any potential bias	2
Interpretation	20	Give a cautious overall interpretation of results considering objectives, limitations, multiplicity of analyses, results from similar studies, and other relevant evidence	N/A
Generalisability	21	Discuss the generalisability (external validity) of the study results	N/A
**Other information**			
Funding	22	Give the source of funding and the role of the funders for the present study and, if applicable, for the original study on which the present article is based	16

*Give information separately for cases and controls in case-control studies and, if applicable, for exposed and unexposed groups in cohort and cross-sectional studies.

Note: An Explanation and Elaboration article discusses each checklist item and gives methodological background and published examples of transparent reporting. The STROBE checklist is best used in conjunction with this article (freely available on the Web sites of PLoS Medicine at http://www.plosmedicine.org/, Annals of Internal Medicine at http://www.annals.org/, and Epidemiology at http://www.epidem.com/). Information on the STROBE Initiative is available at www.strobe-statement.org.

For peer review only

For peer review only - http://bmjopen.bmj.com/site/about/guidelines.xhtml
